# Supplementary material for: Elastic forces drive nonequilibrium pattern formation in a model of nanocrystal ion exchange
Source: Proc Natl Acad Sci U S A. 2021 Dec 21;118(52):e2114551118. doi: 10.1073/pnas.2114551118 (PMC8719903; doi:10.1073/pnas.2114551118)
Supplement: Supplementary File [file pnas.2114551118.sapp.pdf]

PNAS
www.pnas.org

Supplementary Information for

Elastic Forces Drive Nonequilibrium Pattern Formation in a Model of Nanocrystal Ion Exchange

Layne B. Frechette, Christoph Dellago, and Phillip L. Geissler

Christoph Dellago and Phillip L. Geissler.
E-mail: christoph.dellago@univie.ac.at; geissler@berkeley.edu

This PDF file includes:

Fig. S1

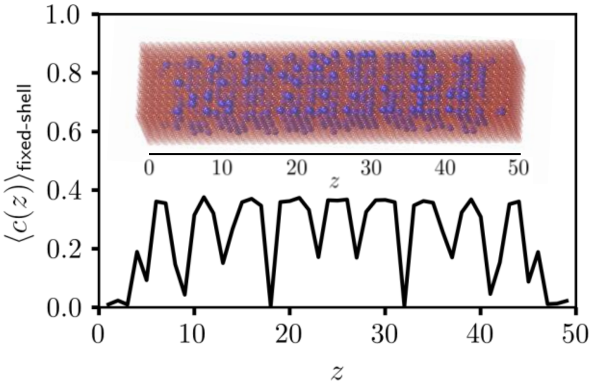

Fig. S1. Average composition profile of an equilibrium core/shell nanorod. The core has dimensions $9 \times 9 \times 49$. The shell is a single layer thick, and $K_{\text{shell}} = 10$. The net composition of the core is fixed at $c = 0.25$, and that of the shell is fixed at $c = 0$. The fixed-shell average was taken over 100 equilibrium configurations. Shell sites were excluded in the composition profile calculation. Inset shows a typical equilibrium configuration, with a scale bar to facilitate comparison with $\langle c(z) \rangle_{\text{fixed-shell}}$.
